# Who is more likely to be obese or overweight among siblings? A nationally representative study in rural China

**DOI:** 10.1371/journal.pone.0187693

**Published:** 2017-11-27

**Authors:** Jiajin Hu, Ning Ding, Shihan Zhen, Yang Liu, Deliang Wen

**Affiliations:** 1 School of Public Health, China Medical University, Shenyang, Liaoning, China; 2 Research Center of Medical Education, China Medical University, Shenyang, Liaoning, China; TNO, NETHERLANDS

## Abstract

**Background:**

The aims of this study were to determine the association between sibling rank and childhood obesity among children ≤ 5 years of age in rural China, and to investigate the effect of child gender and the obesity status of other siblings on this association.

**Methods:**

Data from the China Family Panel Studies, a nationally representative survey, was used for the analysis. Sibling rank was defined as the birth order of all children with the same biological mother. A total of 1116 children ≤ 5 years of age were divided into four groups: children without siblings, first-born children, second-born children, and third-born or younger children. For each child, the body mass index and standard deviation (BMI z score) was calculated according to WHO standards; children with BMI z scores > 2 were classified as obese or overweight (ObOw). Logistic regression models were used to estimate the association between sibling rank and ObOw status, and the possible influence of gender and ObOw status among other siblings.

**Results:**

The second and third-born or younger children had a significantly higher risk of becoming ObOw than children without siblings (odds ratio [OR]: 1.32, 95% confidence interval [CI]: 1.07–1.63 and OR:1.38, 95% CI: 1.17–1.63, respectively). Specifically, female second-born children and male third-born or younger children had a significantly higher risk of ObOw (OR: 1.50, 95% CI: 1.11–2.01 and OR: 1.57, 95% CI: 1.07–2.32, respectively). Having an ObOw sibling increased the probability of being ObOw and the magnitude of the effect was larger if siblings were younger.

**Conclusions:**

Sibling rank was shown to be associated with ObOw status among children 0–5 years of age in rural China. Our findings can help healthcare practitioners and authorities to identify children at risk of obesity. Future studies should focus on the mechanisms of this association.

## Introduction

Over recent decades, the grate of obesity and overweight children has increased significantly on a global scale, particularly in developing countries [1, 2]. In China, the prevalence of obesity and overweight preschool children is 7.2% and 19.8%, respectively [3]. This alarming trend has attracted public and academic attention because childhood obesity is considered a chronic disease [4, 5], and it may be a cause of other chronic diseases in adulthood, such as cardiovascular conditions [6, 7], metabolic syndrome [8], diabetes [9] and asthma [10] and other ailments.

Frequently, there is a clustering of childhood obesity within a family in which the family environment may play a crucial role, including similar unhealthy dietary behaviors and exercise patterns [11–15]. The family environment is particularly influential for preschool children who tend to mimic the behavior of older family members, particularly older siblings. Thus, an investigation in the association between sibling rank and risk of obesity can help health authorities target high-risk populations and shed light on the design of effective and efficient prevention and intervention strategies. Some studies have demonstrated that last-born children are more likely to be obese or overweight (ObOw) [16–20], possibly because younger children may have a higher maternal age at birth, which could be a factor in childhood ObOw [17]. Mothers with larger family sizes can be at higher risk for depression[21], which has been associated with childhood obesity[20]. Also, younger children are more likely to have unhealthy diets than older children [12]. Other studies have failed to find any significant differences, or even the opposite results [22, 23].

Furthermore, most previous studies have not examined the potential effect of gender on the association between sibling rank and obesity, with several studies showing no significant differences.[17, 19, 24]. Also, childhood obesity may be significantly influenced by sibling weight because siblings from the same family may have common risk factors, such as genetic background, socioeconomic status, nutrition intake and physical activity behaviors. No previous studies have focused on the association between sibling rank and childhood ObOw status in China, a country which is experiencing a second-baby boom now that China’s family planning policy has ended [25].

The aims of this study were twofold: (1) to determine the association between sibling rank and ObOw status among Chinese children ≤ 5 years of age, and (2) to investigate how gender and ObOw status of other siblings affects this association further. The results may be useful for health authorities in China and other countries for the design of effective and efficient intervention strategies against childhood obesity.

## Methods

### Data source and participants

The China Family Panel Study (CFPS) is an ongoing nationwide longitudinal social study, which includes 25 of 34 provinces and municipalities and covers 95% of the population of China. The study collected individual, family, and community data; a total of 14,960 families were interviewed. The methodology of this survey has been described elsewhere [26].The study was approved by the Research and Ethical Committee of Peking University (IRB00001052-14010).

We used the data from the first wave of CFPS (2010) which provided a total of 1686 children ≤ 5 years of age and their families from rural areas. The study excluded 326 children because of incomplete data on sibling rank, height, or weight, and it also excluded 244 children in whom the absolute value of BMI z score was > 5. Thus, our sample consisted of 1116 children.

### Sibling rank

In the present study, sibling rank was defined as the birth order of all the children with the same biological mother. It is possible that children from the same household but with different biological mothers had the same sibling rank. Children < 5 years of age or > 5 years of age were included in the sibling rank, even though we focused on children < 5 years of age in the present study. All the children were divided into four groups: children without siblings; first-born children; second-born children; and third-born or younger children. The third-born or younger child group consisted of the third child of the family and his/her younger brothers/sisters (e.g., the fourth or fifth-born child in the family, if any).

### Overweight and obesity

The body mass index (BMI) of a child was calculated on the height (or length) and weight reported by the caregiver. Then, BMI z scores were calculated according to gender and age-specific WHO child growth standards for children between 0 and 5 years of age. Obesity or overweight was defined as a BMI z score > 2.

### Measurement of confounding factors

Child birthweight was based on parent recall. Child ethnicity was provided by the parents and categorized as Han nationality versus others. The duration of breastfeeding was determined by asking parents how many months the children were breastfed. Gestational age was based on self-recall. Maternal age at delivery was calculated by subtracting the age of the child from the age of the mother. The health status of the child was categorized into the following three groups: good (0–1 physician visits in the last year); fair (2–4 visits in the last year); and poor (> 4 visits in the last year). An ObOw sibling was defined as at least one other sibling in the family with a BMI z score > 2. The level of maternal and paternal education was self-reported and categorized into no schooling, completed primary school, completed middle school, and completed high school or above. The caregiver of the child was defined as the person who took care of the child for most of the time during the last month, which was not a vacation month, and separated into three groups: parents, grandparents, and others. Parental emotional support was estimated by asking if the parents actively communicated with the child; the responses were categorized into three groups: positive (strongly agree or agree); neutral (fair), and negative (disagree or strongly disagree).

### Statistical analysis

Logistic regression models were used to quantify the association between sibling rank and ObOw status and and also to investigate the effect of child gender and the obesity status of other siblings on this association. In Model 1, we constructed a categorical variable indicating rank (dummy variables for *the first-born child*, *the second-born child*, and *the third-born or younger child;* reference category = *the child without siblings)*. Also, the difference between the child without siblings and the first-born child was distinguished and the possible non-linearity in the association between sibling rank and ObOw was accounted for. In Model 2, gender linkage was demonstrated by dividing further each of the sibling ranks into two groups (male and female). Finally, in Model 3, we tested the existence of an ObOw link between siblings by dividing each sibling group into two groups (with or without ObOw siblings). In the present study, all estimations of standard errors were corrected by allowing the possible correlation in the ObOw status among Children from different regions of China (e.g. North-East, East, Central and West).

The confounders were controlled in a step-by-step manner. Specification 1 was a crude model (i.e., no controls). Specification 2 controlled for a series of the following individual characteristic covariates: gender (a dummy variable for *males*; reference category = females); age (in years) and its quadratic term; ethnicity (a dummy variable for minority ethnic groups; reference category = the Han ethic group); birthweight (dummy variables for low birthweight [≤ 2.5 kg] and high birthweight [≥ 4 kg]; reference category = normal birthweight [between 2.5 and 4 kg]); duration of breastfeeding (in months); gestational age (in months); maternal age at delivery (in years), and health status of the child (dummy variables for good and poor; reference category = fair). Variables within the household were introduced into Specification 3, which further controlled for the level of education attained by the mother and father (dummy variable for primary school, middle school, high school or above; reference category = no schooling); household income per capita (in 1000 RMB Yuan); the child’s caregiver, and parental emotional support (dummy variables for positive and negative; reference category = neutral).

There were very few missing values for the confounding variables, including birthweight (6.5% missing values), ethnicity (0.4%), duration of breastfeeding (0.8%), gestational age (0.7%), level of education attained by the mother (1.3%), level of education attained by the father (0.6%), and household income per capita (0.1%). In order to make use of the entire sample, we used a multiple imputation technique to fill in ten missing values, which may have been insufficient, as suggested by a previous study [27]. A P value<0.05 was regarded as statistically significant. All analyses were performed using Stata SE (version 13.0).

## Results

The summary statistics in Table 1 present the characteristics of respondents according to sibling rank: 519 children without siblings (46.51%); 81 first-born (eldest) children (7.26%); 440 second-born children (39.43%); and 76 third-born or younger children (6.81%). The ObOw rate of second and third-born or younger children were 39.47% and 35.00%, respectively; only 22.32% of first-born children were ObOw. The findings indicated that first-born children were prone to have ObOw siblings. One-third of first-born children had at least one ObOw sibling compared to 6.59% for second-born children.

**Table 1:** Sample characteristics for the entire sample by birth order of children.

Characteristics	Child without siblings (n = 519, 46.51%)	First-born child (n = 81, 7.26%)	Second-born child (n = 440, 39.43%)	Third-born or younger child (n = 76, 6.81%)
**Age of child (years), M (SD)**	2.47 (1.44)	3.40 (1.21)	2.78 (1.53)	2.82 (1.51)
**Gender, n (%)**				
Male	272 (52.41)	37 (45.68)	248 (56.36)	49 (64.47)
Female	247 (47.59)	44 (54.32)	192 (43.64)	27 (35.53)
**ObOw status, n (%)**				
Normal	375 (72.25)	64 (79.01)	286 (65.00)	46 (60.53)
ObOw	144 (27.75)	17 (20.99)	154 (35.00)	30 (39.47)
**Birthweight, n (%)**^a^				
Low birthweight	29 (5.78)	3 (3.90)	22 (5.49)	8 (12.50)
Normal birthweight	462 (92.03)	69 (89.61)	358 (89.28)	53 (82.81)
Macrosomia	11 (2.19)	5 (6.49)	21 (5.24)	3 (4.69)
**Ethnic groups, n (%)**^b^				
Han	468 (90.52)	70 (86.42)	392 (89.29)	60 (80.00)
Others	49 (9.48)	11 (13.58)	47 (10.71)	15 (20.00)
**Breastfeeding duration(month), M (SD)**^c^	9.98 (6.80)	10.42 (6.60)	11.32 (6.81)	10.84 (6.76)
**Gestational age(month), M (SD)**^d^	9.30 (0.58)	9.29 (0.51)	9.28 (0.62)	9.24 (0.56)
**Maternal age (years), M(SD)**	24.63 (4.08)	24.13 (3.58)	30.50 (4.63)	32.33 (4.58)
**Child’s health status, n (%)**				
Good	229 (44.12)	36 (44.44)	226 (51.36)	46 (60.53)
Fair	172 (33.14)	19 (23.46)	106 (24.09)	10 (13.16)
Bad	118 (22.74)	26 (32.10)	108 (24.55)	20 (26.32)
**Having any ObOw sibling, n (%)**		27 (33.33)	29 (6.59)	13 (17.11)
**Mother's educational attainment, n (%)**^e^				
No schooling	35 (6.90)	17 (20.99)	101 (23.11)	34 (44.74)
Primary school	110 (21.70)	16 (19.75)	133 (30.43)	21 (27.63)
Middle school	291 (57.40)	41 (50.62)	184 (42.11)	18 (23.68)
High school or above	71 (14.00)	7 (8.64)	19 (4.35)	3 (3.95)
**Father's educational attainment, n (%)**^f^				
No schooling	24 (4.67)	8 (9.88)	53 (12.10)	24 (31.58)
Primary school	100 (19.46)	19 (23.46)	134 (30.59)	19 (25.00)
Middle school	295 (57.39)	45 (55.56)	203 (46.35)	29 (38.16)
High school or above	95 (18.48)	9 (11.11)	48 (10.96)	4 (5.26)
**Children carer, n(%)**				
Parents	301 (58.00)	52 (64.20)	326 (74.09)	57 (75.00)
Grandparents	213 (41.04)	26 (32.10)	106 (24.09)	18 (23.68)
Others	5 (0.96)	3 (3.70)	8 (1.82)	1 (1.32)
**Parental emotional support, n (%)**				
Positive	301 (58.00)	43 (53.09)	249 (56.59)	30 (39.47)
Neutral	197 (37.96)	30 (37.04)	169 (38.41)	35 (46.05)
Negative	21 (4.05)	8 (9.88)	22 (5.00)	11 (14.47)
**Family income per capita (1000RMB), M (SD)**^g^	6.67 (10.85)	5.93 (6.81)	7.18 (12.06)	6.17 (7.98)

Note

a: n = 1044 cause of the missing data

b: n = 1112 cause of the missing data

c: n = 1107 cause of the missing data

d: n = 1108 cause of the missing data

e: n = 1101 cause of the missing data

f: n = 1109 cause of the missing data

g: n = 1115 cause of the missing data. Abbreviation: ObOw, obesity or overweight

The results of Model 1 (Table 2) show that, compared to a child without siblings, there was a lower likelihood of ObOw in a first-born child, although it was not significant (OR: 0.69, 95% CI: 0.33–1.44). However, the second-born child and the third-born or younger child had a higher probability of ObOw (OR:1.40, 95% CI: 1.38–1.43 and OR:1.70, 95% CI: 1.28–2.25, respectively). The introduction of confounders decreased the estimated magnitude, but the pattern remained the same (OR:1.32, 95% CI: 1.07–1.63 and OR:1.38, 95% CI: 1.17–1.63, respectively).

**Table 2:** Obesity or overweight status of the children by sibling rank.

	ObOw (BMI z score >2)					
	Specification 1^a^		Specification 2 ^b^		Specification 3 ^c^	
Sibling rank^d^	OR	95%CI	OR	95%CI	OR	95%CI
First-born child	0.69	0.33–1.44	0.71	0.35–1.44	0.66	0.31–1.41
Second-born child	1.40	1.38–1.43	1.45	1.25–1.67	1.32	1.07–1.63
Third-born or younger child	1.70	1.28–2.25	1.73	1.42–2.11	1.38	1.17–1.63

a: No adjustment was performed

b: Adjusted for gender, birthweight, health status of the child, duration of breastfeeding, maternal age at delivery, gestational age, age of child, child age squared, ethnic group

c: Adjusted for gender, birthweight, health status of the child, duration of breastfeeding, maternal age at delivery, gestational age, age of child, child’s age squared, ethnic group, level of education attained by the mother, level of education attained by the father, family income per capita, child’s caregiver, parental emotional support

d: Reference: Child without siblings

For the second-born child, the results of Model 2 (Table 3) showed that the probability of female ObOw (OR: 1.50, 95% CI: 1.11–2.01) was significantly higher than the reference group, while for the third-born or younger child, males had a higher probability of ObOw (OR: 1.57, 95% CI: 1.07–2.32). The probability of ObOw in the male second-born child and the female third-born or younger child were not statistically significant compared with the reference group. The probability of ObOw in the first-born male and female children was lower compared to the reference group, but not statistically significant (OR:0.72, 95% CI: 0.34–1.56 and OR:0.56, 95% CI: 0.17–1.81, respectively).

**Table 3:** Association between sibling rank and childhood obesity or overweight by gender.

Sibling rank	ObOw (BMI z score >2)			
	Male^a^^.^^c^		Female^b^^.^^c^	
	OR	95% CI	OR	95% CI
First-born child	**0.72**	**0.34–1.56**	**0.56**	**0.17–1.81**
Second-born child	**1.22**	**0.87–1.71**	**1.50**	**1.11–2.01**
Third-born or younger child	**1.57**	**1.07–2.32**	**1.16**	**0.59–2.27**

a:Reference: a male child without siblings

b:Reference: a female child without siblings

c: Adjusted for birthweight, health status of the child, duration of breastfeeding, maternal age at delivery, gestational age, age of child, child’S age squared, ethnic group, level of education attained by the mother, level of education attained by the father, family income per capita, caregiver of the child, parental emotional support.

Table 4 demonstrates the influence of sibling ObOw status on a child. If the second-born child did not have ObOw siblings, the probability of ObOw was 1.23 (95% CI:1.03–1.46), whereas if the second-born child had ObOw siblings, the probability of ObOw increased significantly (OR = 2.59, 95% CI:1.27–5.30). For the third-born or younger children, the probability of having an ObOw sibling was even higher (OR = 4.23, 95% CI:1.32–3.84); however, if there were no other ObOw siblings, the probability of being obese or overweight was not statistically significant (OR:1.06, 95% CI: 0.80–1.42)

**Table 4:** Association between birth order, having an ObOw sibling and childhood obesity or overweight.

	ObOw (BMI z score > 2) ^b^	
	OR	95%CI
Sibling rank by having or not ObOw siblings ^a^		
First-born child without ObOw siblings	0.51	0.19–1.36
First-born child with ObOw siblings	1.01	0.62–1.65
Second-born child without ObOw siblings	1.23	1.03–1.46
Second-born child with ObOw siblings	2.59	1.27–5.30
Third-born or younger child without ObOw siblings	1.06	0.80–1.42
Third-born or younger child with ObOw siblings	4.23	1.32–13.61

a:Reference: a child without siblings

b: Adjusted for gender, birthweight, health status of children, duration of breastfeeding, maternal age at delivery, gestational age, age of child, child’s age squared, ethnic group, level of education attained by the mother, level of education attained by the father, family income per capita, caregiver of the child, parental emotional support.

## Discussion

Based on the data from a nationally representative survey in China, our study findings suggest that sibling rank is significantly associated with obesity or overweight status of children < 5 years of age. Specifically, compared to children without siblings, lower sibling rank had a higher risk of ObOw. The association may be influenced by the gender of the child. Female second-born children and male third-born or younger children were more likely to be ObOw. Children with an ObOw sibling were more likely to be ObOw.

Our study has contributed to the literature by demonstrating a significant association between sibling rank and childhood obesity. However, it is reasonable to propose that the association may be biased because of the existence of many confounders. For example, parents with more than one children usually have lower education attainment in China which can lead to childhood obesity or overweight. Thus, sibling rank is a surrogate of parental education. Another example is birthweight which, as confirmed, can increase the risk of childhood ObOw [21]. The findings from our sample suggest that younger siblings had a higher probability of low birthweight. however, after introducing these two confounders, the significance of estimates remain, suggesting other mechanisms. Parental preference may play an important role because when faced with a limited family budget parents may prioritize the youngest children with respect to nutrition and energy intake. Also, it is possible that older children among siblings may help their parents take care of their younger siblings and may consume extra calories [28, 29], particularly among children > 2 years of age [30].

The results showed that children with ObOw siblings have a higher probability of ObOw, possibly because siblings share the similar genetic background and they are likely to have a similar apparent phenotype, such as body height [31] and weight [32]. Siblings are likely to have similar lifestyles, including food preferences [13, 14] and involvement in physical activity [14, 15]. Also, it is possible that younger children imitate the behaviors of older siblings[29].

Unlike the study of Mosli *et al*. [24], we identified a significant gender difference in the association between sibling rank and ObOw status. A previous study [3] reported that boys are more likely to be ObOw than girls in China; however, our study suggests that the female second-born child and the male third-born or younger child had a higher probability of ObOw. The mechanisms underlying these associations have not been established, and they warrant further study.

Our study had several limitations. First, the key anthropometric measurements of the present study (i.e., weight and height or length) are proxy-reported. Admittedly, self-reporting error may bias estimations; however, if report error is not systematically related to the birth order or gender mix of children, such an error would not influence the estimates significantly [12]. Furthermore, if reporting error is constant within each household (i.e., the same individual reports the length / height and weight for each child in a household), the bias in estimated correlation may not be significant. Second, the information on the number of ObOw siblings may not be accurate because older siblings (> 15 years of age) leave home, and therefore they may fall out of the sample framework as they grow up. This phenomenon may be uncommon because in such circumstances the age difference between siblings is likely to be > 10 years. Third, although various confounders were considered in this study, there were some potential socioeconomic and genetic factors that could confound the estimates. Limited by variables that collected and the sample size, we were unable to explain the mechanisms underlying the association between sibling rank and ObOw, nor how gender and an ObOw sibling further affect the association. In further studies, it will be beneficial to focus on ObOw behaviors among siblings within the same family to explore the underlying mechanisms. Finally, our findings may not be generalized to the entire population because our sample was restricted to children < 5 years of age. It remains unclear if the association between sibling rank and obesity status persists into later life.

## Conclusions

Our study showed that sibling rank is associated with ObOw status among children < 5 years of age in rural China. Younger siblings are more likely to be ObOw than children without siblings. There were gender differences in the association between sibling rank and childhood ObOw status. Children with other ObOw siblings were more likely to be ObOw. Our findings can help healthcare practitioners and authorities identify children at risk of obesity and design prevention and intervention policies.

## Supporting information

S1 Appendix PRISMA statement. (DOC)

S2 Appendix The raw data of the present study. https://figshare.com/s/4b4094aacff15b0ea90a. (DTA)
